# Isoliquiritigenin Suppresses E2-Induced Uterine Leiomyoma Growth through the Modulation of Cell Death Program and the Repression of ECM Accumulation

**DOI:** 10.3390/cancers11081131

**Published:** 2019-08-07

**Authors:** Po-Han Lin, Hsiang-Lin Kung, Hsin-Yuan Chen, Ko-Chieh Huang, Shih-Min Hsia

**Affiliations:** 1 School of Nutrition and Health Sciences, College of Nutrition, Taipei Medical University, Taipei 11031, Taiwan; 2 Graduate Institute of Metabolism and Obesity Sciences, College of Nutrition, Taipei Medical University, Taipei 11031, Taiwan; 3 School of Food and Safety, Taipei Medical University, Taipei 11031, Taiwan; 4 Nutrition Research Center, Taipei Medical University Hospital, Taipei 11031, Taiwan

**Keywords:** uterine leiomyoma, isoliquiritigenin, apoptosis, autophagy, extracellular matrix

## Abstract

Uterine leiomyomas, also known as fibroids, are common and prevalent in women of reproductive age. In this study, the effect of Isoliquiritigenin (ISL), a licorice flavonoid, on the anti-proliferation of uterine leiomyoma was investigated. We found that the survival of uterine leiomyoma ELT3 cells and primary uterine smooth muscle (UtSMC) cells was reduced by treatment with ISL alone or with ISL plus estradiol (E2). Cell cycles were arrested through the reduction of G2/M- and S-phase populations in ELT3 and UtSMC cells, respectively. Furthermore, increased sub-G1 phase and nucleus condensation were observed in ELT3 cells but not in UtSMC cells. Co-treatment of ELT3 cells with E2 and ISL inhibited ERK1/2 activation, whereas p38 and c-Jun N-terminal kinase (JNK) activation was enhanced. Moreover, ISL-induced apoptosis and autophagy cell death in ELT3 cells were observed. Serum E2 and P4 levels were reduced in a E2-enhanced uterine myometrium hyperplasia mouse model by ISL treatment, which contributed to the downregulation of the expression of extracellular matrix (ECM) associated proteins and matrix metalloproteinase (MMPs). Taken together, these results showed that ISL exerted a higher effect on the inhibition of estrogen-induced uterine leiomyoma growth for both in vitro and in vivo ECM accumulation, demonstrating its potential as a new option for treatment of uterine leiomyoma.

## 1. Introduction

Benign uterine leiomyomas, also known as fibroids or myomas, are one of most common female reproductive disorders found in women of reproductive age [1]. These leiomyomas initially develop from the smooth muscle hyperproliferation of the uterus. The risk factors include age, obesity, premenopausal status, race/ethnicity, and lifestyle [2,3,4]. Epidemiologic data show that African-American women generally develop uterine leiomyomas at 25 years of age, which is earlier than Caucasian women by 10 years, indicating a higher incidence in blacks than in whites. By 50 years of age, the prevalence is 70% to 80% [5,6,7]. Women with uterine leiomyomas can be asymptomatic or symptoms develop insidiously. However, only 20% to 30% of these women show symptoms of uterine leiomyomas [8]. The clinical features of women with uterine leiomyomas include severe menstrual bleeding, pelvic pressure and pain, increased urinary frequency, and infertility [9,10,11]. The management of uterine leiomyoma strictly depends on several factors, including location, number, and size. Nevertheless, surgical therapies, such as myomectomy and hysterectomy, have long been the mainstay of uterine leiomyomas treatment. For example, submucous myomas have been indicated to impact future embryo implantation. Reportedly, submucous myomas surgically excised by a Hysteroscopic Tissue Removal systems operation had a good outcome, especially on reducing the operating times and complications after surgery [12,13]. However, surgical therapies are associated with a high risk of morbidity, mortality [14], and the risk of recurrence [15], as well as an increased economic burden [16]. Although most women with uterine leiomyoma have a high chance of regular pregnancy, this is accompanied by a high risk of obstetric complications, such as spontaneous miscarriage, preterm labor, or placental abruption [17,18]. The size of uterine leiomyoma during pregnancy and puerperium still exerts not positive modification or improvement [19]; however, routine myomectomy in pregnant women is not recommended [18]. Women who wish to retain their uterus for further pregnancies or other reasons do not accept surgical therapies. Hence, developing other options for treatment and prevention of uterine leiomyola is necessary.

Plant-based medicines have been investigated as a therapeutic strategy for cancer treatment [20]. The roots and rhizomes of licorice, *Glycyrrhiza uralensis*, have been widely used as a natural sweetener and serve as a traditional Chinese medicine (TCM) in Asian countries. Isoliquiritigenin (ISL), 2′,4,4′,-trihydroxychalcone, is among the flavanone components that exist in the roots of licorice and is also found in *Dianthus chinensis, Astragalusmembranaceus* (Fisch.) Bunge, *Dalbergia odorifera*, shallots (*Liliaceae*), and bean sprouts [21,22]. ISL has been reported to exert various biological properties, including antioxidant [23] and anti-inflammatory [24] activities, anti-diabetic activity for the improvement of glucose tolerance [25], cardioprotection against ischemic injury [26], neuroprotection against Parkinson’s disease, and anti-Alzheimer’s activity [27,28,29]. ISL can effectively reduce cell growth of colon, lung, and prostate cancers [30,31,32]. ISL and its derivatives have been reported to inhibit oral cancer proliferation [33,34].

The effects of ISL on gynecologic cancers have been highly attended. We previously reported that ISL treatment can suppress breast cancer cell migration [35] and inhibit ovarian cancer cell proliferation through activating autophagy process signaling [36]. Our animal study showed that endometrial cancer growth is inhibited by ISL treatment via inducing apoptosis and trigging autophagic-related signaling activation [37]. Recently, we found that ISL can enhance doxorubicin chemosensitivity on the inhibition of uterine sarcoma cell growth [38]. However, the efficiency of ISL on the inhibition of uterine leiomyoma growth is still unclear.

The pathogenesis of uterine leiomyoma is complex and involves aberrant genetic and epigenetic changes of hormones, growth factors, and chromosomal characteristics [39,40]. Excessive estrogen levels have been reported to promote the growth of uterine leiomyoma by influencing growth factors and its signaling pathway [39,41]. In this study, we evaluated the effect of ISL on estrogen-induced uterine leiomyoma and normal uterine myometrial cell growth and examined the molecular mechanisms underlying ISL-induced proliferation inhibition of uterine leiomyoma in vitro and in vivo. Our results demonstrated that ISL had a higher selective capacity on inhibiting uterine leiomyoma cell proliferation by inducing apoptosis and autophagy signaling activation. ISL treatment can tightly control normal uterine myometrial cell growth by arresting cell cycle progression. The findings of this study provide valuable insights for an option for treatment on uterine leiomyoma.

## 2. Results

### 2.1. Inhibition of Cell Viability in ELT3 and Uterine Smooth Muscle (UtSMC) Cells by ISL

**Figure 1:**
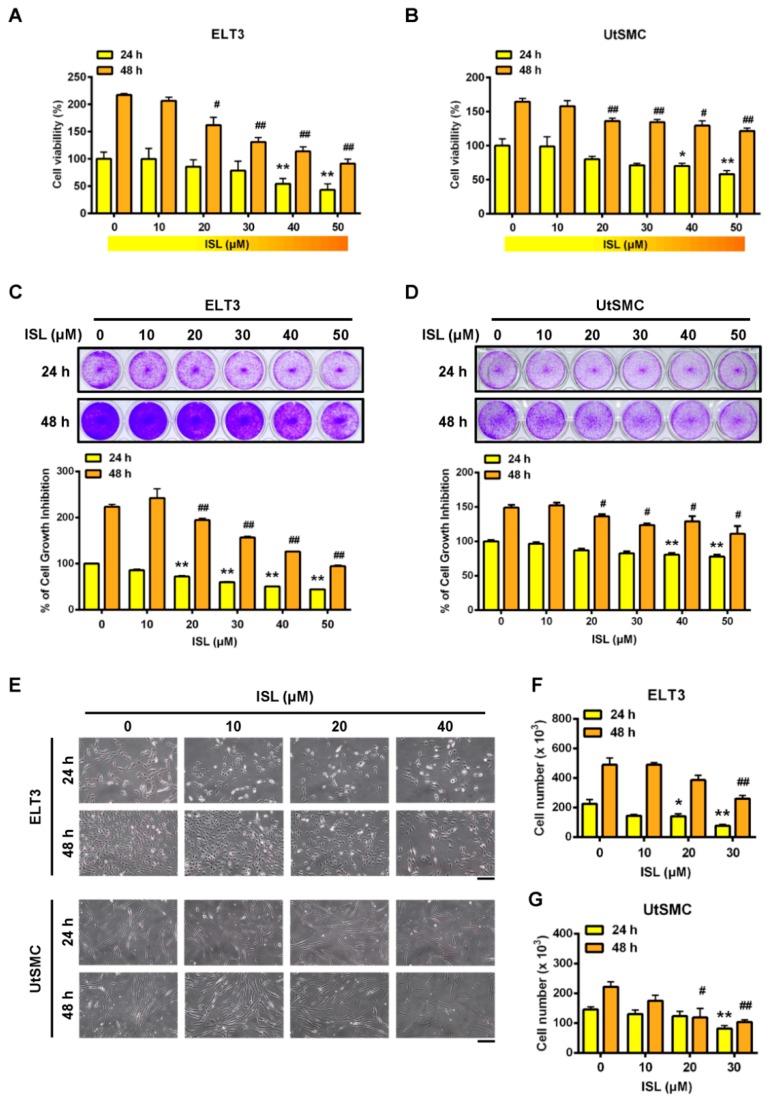
Effects of Isoliquiritigenin (ISL) on the cell viability of uterine leiomyoma ELT3 cells and primary uterine smooth muscle (UtSMC) cells. (**A**,**B**) ELT3 and UtSMC cells were seeded in 96-well plates (2500 cells per well) with 100 μL per well culture medium. After cell attachment to the bottom of well, both cell types were treated with ISL in various doses for 24 and 48 h. At the end of incubation, cell viability was measured by the MTT assay (*n* = 4). (**C**,**D**) ELT3 (1.8 × 10^4^ cells per well) and UtSMC (2.5 × 10^4^ cells per well) cells were seeded in 24-well plates. Both cell types were treated with various doses of ISL for 24 and 48 h. Cell viability was detected using the crystal violet assay (*n* = 4). (**E**–**G**) ELT3 (6 × 10^4^ cells per well) and UtSMC (1.5 × 10^5^ cells per well) cells were seeded in the 6-well plates. Both cell types were treated with ISL in various doses for 24 and 48 h. Cell morphology was photographed and cell numbers were counted using trypan blue stain and an automatic cell counter (*n* = 3). (magnification 100×; Scale bar = 20 μm). Data are represented as means ± SEM. * *p* < 0.05, ** *p* < 0.01 compared with the 24 h-control group. ^#^ *p* < 0.05, ^##^ *p* < 0.01 compared with the 48 h-control group.

Initially, to evaluate whether ISL has the ability to inhibit cell proliferation in uterine leiomyoma ELT3 and UtSMC cells were treated with serial doses of ISL (10, 20, 30, 40, and 50 μM) for 24 and 48 h. The results showed that treatment of ELT3 cells with ISL at a concentration above 40 μM for 24 h inhibited cell proliferation. When ELT3 cells were treated for 48 h, ELT3 cell proliferation was significantly reduced in the presence of ISL at 20 to 50 μM (Figure 1A). In contrast, treatment of UtSMC cells with ISL for 24 and 48 h had a slight inhibitory effect on cell proliferation, even in the presence of ISL at concentrations higher than 40 μM (Figure 1B). The results of the crystal violet assay aligned with those of the MTT assay were as follows: The inhibitory effect of ISL was observed in both ELT3 and UtSMC cells (Figure 1C,D). In the presence of ISL at 40 μM, a significantly reduced cell number and shrinking, scrawny, and rounded floating cell morphologies were observed in ELT3 cells (Figure 1E, upper panel, and Figure 1F). In addition, a flattened and very slim cell morphology was observed in UtSMC cells (Figure 1E, lower panel). The number of UtSMC cells also slightly reduced by ISL treatment (Figure 1G). These results suggested that ISL presents a potential role in the suppression of uterine leiomyoma growth.

### 2.2. Effects of ISL Treatment on E2-Induced Cell Proliferation in ELT3 and UtSMC Cells

**Figure 2:**
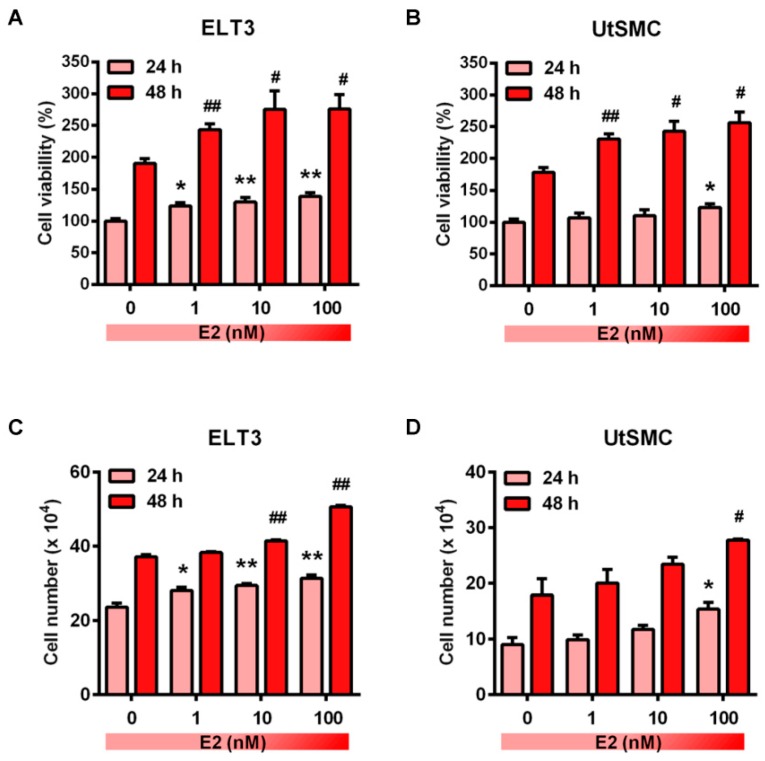
Effects of estradiol on the growth of ELT3 and UtSMC cells. (**A**,**B**) Both ELT3 and UtSMC cells were seeded at 3000 cells per well in 96-well plates. Cells were treated with E2 in serial concentrations for 24 and 48 h. Cell viability was analyzed using the MTT assay (*n* = 4). (**C**,**D**) ELT3 (6 × 10^4^ cells per well) and UtSMC (1.5 × 10^5^ cells per well) cells were seeded in 6-well plates. Cells were treated with serial concentrations of E2 for 24 and 48 h. Cell numbers were counted using trypan blue stain (*n* = 3). Data are represented as means ± SEM. * *p* < 0.05, ** *p* < 0.01 compared with the 24 h-control group. ^#^ *p* < 0.05, ^##^ *p* < 0.01 compared with the 48 h-control group.

**Figure 3:**
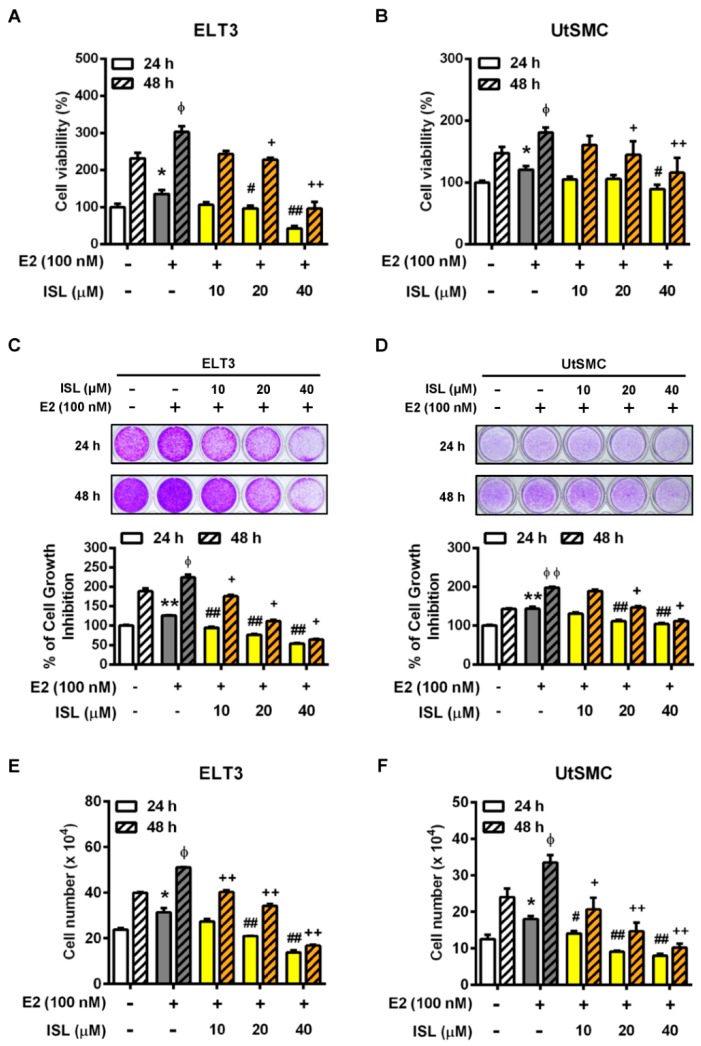
Effects of ISL on the E2-induced cell growth in ELT3 and UtSMC cells. (**A**,**B**) ELT3 and UtSMC cells were seeded at 3000 cells per well in 96-well plates. Both cell types were treated with E2 (100 nM) alone or E2 plus ISL at 10, 20, or 40 μM for 24 and 48 h. Cell viability was detected using crystal violet assay (*n* = 4). (**C**,**D**) ELT3 (6 × 10^4^ cells per well) and UtSMC (1.5 × 10^5^ cells per well) cells were seeded in 6-well plates. Both cell types were treated as indicated above. Cell viability was detected using crystal violet assay (*n* = 4). (**E**,**F**) ELT3 (6 × 10^4^ cells per well) and UtSMC (1.5 × 10^5^ cells per well) cells were seeded in 6-well plates. Cells were treated as indicated above. Cell numbers were counted using trypan blue stain (*n* = 3). Data are represented as means ± SEM. * *p* < 0.05, ** *p* < 0.01 as compared with the 24 h-control group. ^ϕ^ *p* < 0.05, ^ϕϕ^ *p* < 0.01 compared with the 48 h-control group. ^#^ *p* < 0.05, ^##^ *p* < 0.01 compared with the 24 h E2-treated group. ^+^ *p* < 0.05, ^++^ *p* < 0.01 compared with the 48 h E2-treated group.

Sexual steroid hormones have been reported to promote uterine fibroblast growth [42,43]. Specifically, the over-expression level of aromatase p450 was identified in uterine leiomyoma that catalyzes androgens to estrogens in situ and has a critical role in the promotion of leiomyoma growth [44,45]. Therefore, we first identified whether treatment of ELT and UtSMC cells with E2 promoted cell growth. The results showed that the cell proliferation rate of ELT3 and UtSMC cells increased after treatment of cells with E2 at concentrations from 1 to 100 nM for 24 and 48 h (Figure 2A,B). The cell numbers results aligned with those from the MTT assay in both ELT3 and UtSMC cells (Figure 2C,D). Therefore, we further examined whether ISL could inhibit E2-induced ELT3 and UtSMC cell proliferation. The MTT assay results showed that E2-induced cell proliferation was inhibited by co-treatment with ISL in both ELT3 and UtSMC cells (Figure 3A,B). The results of the crystal violet assay and the cell number assay were consistent with MTT assay in both ELT3 and UtSMC cells (Figure 3C–F).

### 2.3. Effects of ISL Treatment on the Cell Cycle Progression of ELT3 and UtSMC Cells

**Figure 4:**
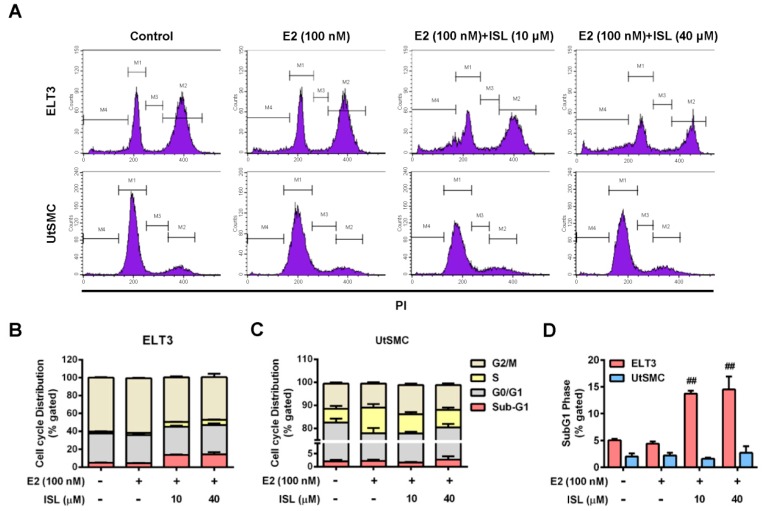

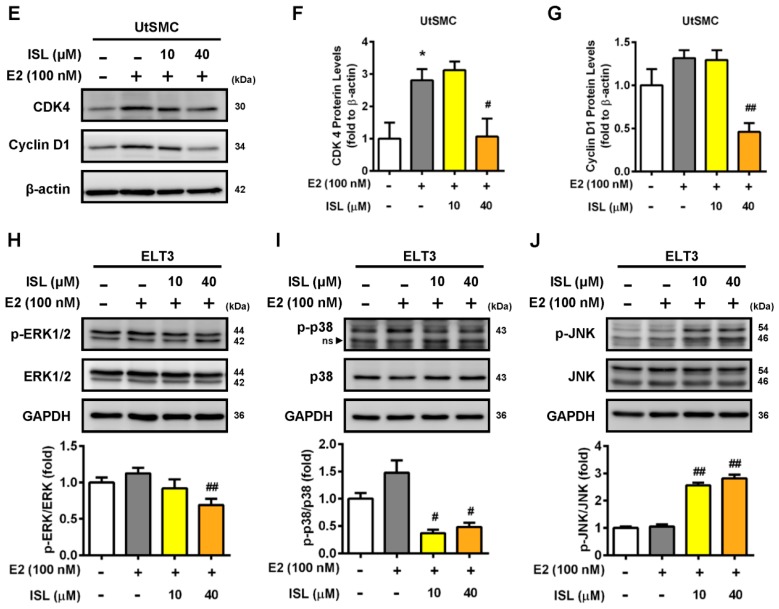
Determine the cell cycle distribution of ELT3 and UtSMC cells by ISL treatment. (**A**) ELT3 and UtSMC cells were treated with E2 (100 nM) alone or a combination of E2 and ISL (10 or 40 μM) for 48 h. Cells were stained with propidium iodide, and the cell cycle distributions were analyzed by flow cytometry. (**B**–**D**) The quantitative data of cell distribution in ELT3 and UtSMC cells was shown. (**E**–**G**) UtSMC cells were co-treated with E2 and ISL for 48 h. The expressions of cyclinD1 and CDK4 protein in UtSMC cells were analyzed using Western blot. Each target protein was normalized to β-actin expression. (**H**–**J**) ELT3 cells were co-treated with E2 and ISL for 48 h. The expressions of MAPK proteins were analyzed using Western blot. Each target protein was normalized to GAPDH expression. Data are represented as means ± SEM. * *p* < 0.05 compared with the control group. ^#^ *p* < 0.05, ^##^ *p* < 0.01 compared with the E2-treated group.

To confirm whether ISL-induced cell proliferation suppression occurs through regulated cell cycle progression, the cells were stained with PI and analyzed using flow cytometry. The results showed that cell cycle progression in ELT3 cells treated with E2 alone was not affected. However, ELT3 cells co-treated with E2 and an ISL concentration of 10 and 40 μM decreased G2/M phases distribution (Figure 4A,B). In contrast, treatment of E2 significantly increased S phase distribution in UtSMC cells. This increased effect was reduced by co-treatment with E2 and ISL (Figure 4A,C). The distribution of the sub-G1 phase increased with co-treatment with E2 and ISL, but this effect was not observed in UtSMC cells (Figure 4D). We further examined whether the expression of cyclin/CDK complex involved in suppression of cell cycle at S-phase by treatment with ISL in UtSMC cells and the expression of cyclin D1/CDK4 complex in UtSMC cells was monitored. The results showed that treatment with E2 increased cyclin D1 expression, but the expression of cyclin D1 and CDK4 protein level were decreased by co-treatment with E2 and ISL at 40 μM for 48 h in UtSMC cells (Figure 4E–G). It has been reported that the mitogen-activated protein kinase (MAPK) pathway is important in cell survival and apoptosis regulation [46]. We further analyzed the expression of MAPK proteins using Western blot. The results showed that co-treatment of cells with E2 and ISL concentration of 10 and 40 μM reduced the expression of phosphorylated extracellular signal-regulated kinase-1 and -2 (ERK1/2) protein (Figure 4H), whereas the expressions of phosphorylated p38 and c-Jun N-terminal kinase (JNK) proteins were increased (Figure 4I,J). Additionally, Hoechst 33342 staining was performed for observation of DNA condensation. The results showed that treatment with E2 alone did not produce any alterations in ELT3 cells; however, co-treatment with E2 and ISL markedly increased the population of DNA-condensed cells, confirmed by the observation of higher strength blue fluorescence. In contrast, we observed no alteration in nuclear stains in UtSMC cells after co-treatment with E2 and ISL (Figure S1).

### 2.4. Effects of ISL Treatment on Apoptosis and Levels of Apoptosis-Associated Proteins in ELT3 and UtSMC Cells

**Figure 5:**
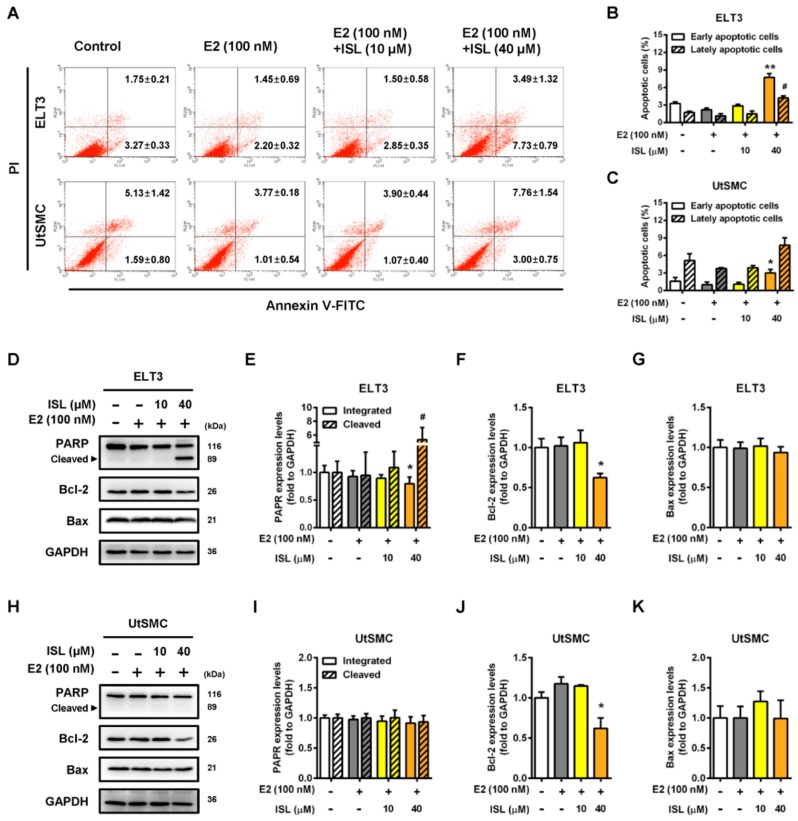
ISL induced cell apoptosis via upregulation of apoptotic associated protein expression in ELT3 cells. (**A**) ETL3 and UtSMC cells were treated with E2 (100 nM) alone or a combination of E2 and ISL (10 or 40 μM) for 48 h. Cells were stained with propidium iodide and Annexin V-FITC, and the apoptosis rates were analyzed by the flow cytometry. (**B**,**C**) The quantitative data of apoptotic cell death in early and late phases are shown. After treatment as indicated above, the apoptotic-associated proteins were analyzed using Western blot in (**D**) ELT3 and (**H**) UtSMC cells. Each target protein was normalized to GAPDH expression in (**E**–**G**) ELT3 and (**I**–**K**) UtSMC cells. Data are represented as means ± SEM (*n* = 3). * *p* < 0.05, ** *p* < 0.01 compared with the E2-treated group. ^#^ *p* < 0.05 compared with the lately apoptotic phase of the E2-treated group or the level of cleaved PARP form in the E2-treated group.

To further confirm whether ISL induced cell apoptosis in ELT3 and UtSMC cells, the cells were double stained with PI and Annexin V-FITC, and the apoptotic cell percentage was analyzed by flow cytometry. The results showed that the percentages of annexin-V-positive in ELT3 cells, indicating early apoptotic status, and PI- and Annexin-V-positive, indicating early apoptotic status, increased by co-treatment with E2 and an ISL concentration of 40 μM compared with the E2 alone treatment group (Figure 5A,B). In contrast, UtSMC cells co-treated with E2 and ISL at 40 μM increased the Annexin-V-positive cell population, but the PI- and Annexin-V-positive cell populations did not change (Figure 5A,C). The apoptosis-associated proteins levels were analyzed by Western blot. We found that co-treatment with E2 and ISL at 40 μM for 48 h decreased the expression levels of total PARP protein in ELT3 cells. Moreover, the expression level of cleaved PARP protein increased by co-treatment with E2 and ISL at 40 μM. The expression level of Bcl-2 protein was lower in ELT3 cells by co-treatment with E2 and ISL, but the Bax protein expression level was not altered (Figure 5D–G). The expression level of Bcl-2 in UtSMC cells was observed to be parallel with ELT3 cells co-treated with E2 and ISL at 40 μM. However, PARP and Bax protein levels were not altered by co-treatment with E2 and ISL in UtSMC cells (Figure 5H–K).

### 2.5. Effects of ISL Treatment on Levels of Autophagy-Associated Proteins in ELT3 Cells

**Figure 6:**
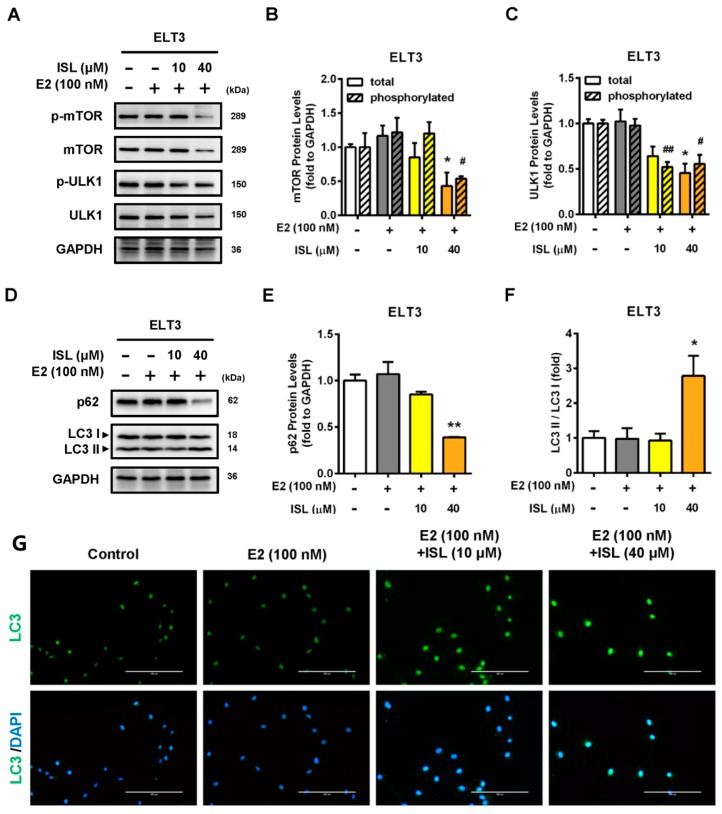
ISL triggered autophagy in ELT3 cells. ELT3 cells were treated with E2 alone or a combination of E2 and ISL (10 or 40 μM) for 48 h. (**A**,**D**) Cells were lysed, and then autophagy-associated proteins were analyzed by Western blot. (**B**–**F**) Each target protein was normalized to GAPDH expression in ELT3 cells. (**G**) The expression of LC3II (green) and DAPI (blue) immunofluorescence of ELT3 cells was evaluated (magnification 200×; Scale bar = 200 μm). Data represented as means ± SEM (*n* = 3). * *p* < 0.05, ** *p* < 0.01 compared with the total protein level of E2-treated group. ^#^ *p* < 0.05, ^##^ *p* < 0.01 compared with the phosphorylated protein level of E2-treated group.

Bcl-2 can prevent the formation of the autophagosome initiation complex, preventing cells from undergoing autophagy [47,48]. Therefore, we further investigated whether downregulation of the Bcl-2 protein expression by ISL could trigger cell autophagy process. ELT3 cells co-treated with E2 and ISL at 40 μM had decreased mTOR total and phosphorylated protein expression levels which in turn reduced ULK1, a downstream signal molecule, and total and phosphorylated protein expression (Figure 6A–C). The expression levels of autophagy marker proteins p62 and LC3 were analyzed. The results showed that the expression of p62 protein was decreased after co-treatment with E2 and ISL at 40 μM for 48 h in ELT3 cells. However, the conversion of LC3 I to LC3 II increased, leading to an increase in the LC3 II/LC3 I ratio (Figure 6D–F). Immunofluorescent staining was performed using anti-LC3 antibody. In parallel, the immunofluorescent staining results showed that co-treatment with E2 and ISL at 40 μM significantly increased LC3 II protein expression (Figure 6G).

### 2.6. Inhibition of E2-Induced Uterine Myometrium Growth by Co-Treatment with ISL In Vivo

**Figure 7:**
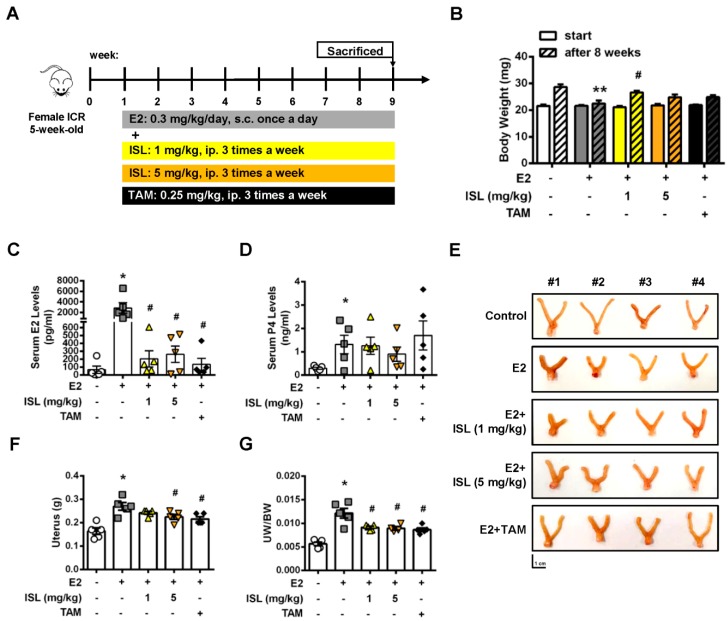
Effects of ISL treatment on E2-induced mouse uterine myometrium growth. (**A**) Schematic design of the in vivo mice uterine myometrium hyperplasia model. (**B**) Mice body weights at the beginning and the end of the study. After eight weeks treatment, mice were sacrificed, and serum (**C**) estradiol and (**D**) progesterone concentrations were measured using ELISA. (**E**) The uterus was photographed, (**F**) the weights of uteri were measured and (**G**) normalized to whole body weight. Scale bar = 1 cm. Data are represented as means ± SEM (*n* = 5 each group). * *p* < 0.05, ** *p* < 0.01 compared with the control group. ^#^ *p* < 0.05 compared with the E2-treated group.

**Figure 8:**
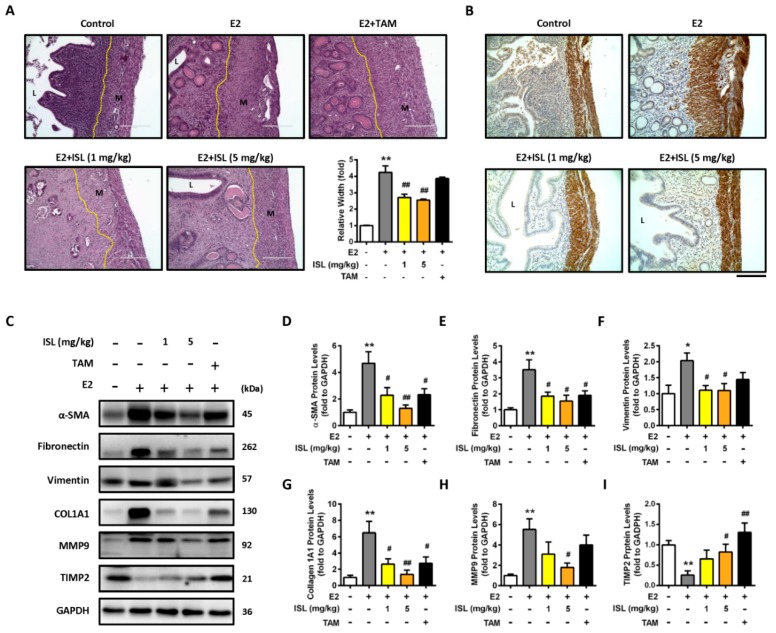
ISL suppressed E2-induced uterine myometrium hyperproliferation and extracellular matrix-associated protein expression. (**A**) To three to five micrometer cross-sections of uterine tissues, hematoxylin and eosin (H&E) staining was performed, and the thick of myometrium layer was evaluated (magnification 200×; Scale bar = 200 μm). (**B**) The expression of smooth muscle target protein was analyzed using immunohistochemical staining of uterine sections with anti-alpha-smooth muscle actin (α-SMC) antibody magnification 200×; Scale bar = 200 μm). (**C**) Uterine tissues were lysed, and extracellular matrix associated proteins were analyzed by Western blot; (**D**–**I**) each target protein was normalized to GAPDH expression. Data are represented as means ± SEM (*n* = 5 each group). * *p* < 0.05, ** *p* < 0.01 compared with the control group. ^#^ *p* < 0.05, ^##^ *p* < 0.01 compared with the E2-treated group. L, luminal space; M, myometrium.

We further investigated whether ISL could inhibit E2-induced uterine myometrium growth in vivo. The mouse model was generated as shown in Figure 7A. The ICR mice were administered E2 alone, or a combination of E2 and ISL or tamoxifen (TAM), as a selective estrogen receptor modulator, for eight weeks. The results showed that mice administered E2 for eight weeks had a lower body weight compared with the control group, and this effect seemed to increase by co-treating mice with E2 and ISL or TAM (Figure 7B). At the end of treatment, the serum levels of E2 and P4 were measured and the weights of uteri were monitored. The serum levels of E2 and P4 increased in the E2-only treatment group compared with the control group. This enhanced effect on the E2 serum level was reduced by co-treating with E2 and ISL at two different concentration groups, or the E2 plus TAM group, but no alteration in the P4 serum level was observed (Figure 7C,D). E2-treatment-increased weight of the uterus was decreased by co-treating with E2 and the low and high dosages of ISL. This effect was also observed in the group co-treated with E2 and TAM (Figure 7E–G). The histopathological analysis was further performed on the uteri tissues. E2 treatment significantly increased uterine myometrium layer, confirmed by the H&E staining assay. This effect was reduced by co-treatment with E2 and ISL at two different concentrations, but co-treatment with E2 and TAM did not have an effect (Figure 8A). The expression of α-SMC protein in the uterine myometrium was detected using immunohistochemistry analysis. Compared with the control group, E2 treatment increased the expression of α-SMC, and this effect was reduced by co-treatment with E2 and ISL (Figure 8B), suggesting that ISL has the potential to inhibit uterine myometrium growth. The excessive deposition of extracellular matrix (ECM)-associated proteins is one characteristic of uterine leiomyoma [49].

In addition, the effects of ISL on the levels of ECM-associated proteins in E2-induced hyperplasic uteri were further evaluated using Western blot assay. Consistently, the result for the α-SMC protein expression level was similar to the immunohistochemistry analysis (Figure 8C,D). The E2-induced enhancement of the expression of fibronectin, vimentin, type I alpha 1 collagen (COL1A1), and MMP9 protein levels was reduced by co-treatment with E2 and ISL. Co-treated mice with E2 and TAM showed the partial reduction ability of ECM-associated protein expression (Figure 8C,E–H). Subsequently, this effect was also evaluated with the ELT3 cell model, and the results showed that the E2-induced expression levels of ECM-associated proteins, including fibronectin, vimentin, and biglycan, decreased by co-treatment E2 and ISL (Figure S2A–D). Opposite results were observed: The E2-treatment-reduced level of TIMP2 protein was increased by co-treatment with E2 and ISL or TAM (Figure 8C,I).

## 3. Discussion

Flavonoids are widely present in plant-based food, nutraceuticals, and traditional Chinese medicine (TCM). They are one of the biologic compound groups in licorice and are known to have strong anticancer activities. Among the flavonoids, quercetin and liquiritin have been shown to exert variable biologic activities, and also showed an inhibitory activity on the growth of various cancer cells via inducing apoptosis progression [50,51,52]. ISL also has anticancer growth activity. Previously, we reported that ISL treatment could inhibit gynecologic cancers’ progression, including breast, ovary, uterine sarcoma, and endometrial cancers [35,36,37,38]. In this study, we uncovered a completely novel role of ISL in anti-proliferation of uterine leiomyoma. We found that ISL treatment could inhibit uterine leiomyoma ELT3 cell growth by arresting the cell cycle and inducing cell apoptosis and autophagy progression. However, normal primary human uterine smooth muscle (UtSMC) cell growth was suppressed by ISL treatment at a higher concentration by reducing the cell cycle in the S phase, but cells did not undergo further apoptosis or autophagy in the cell death program. These results suggest that ISL exerts a highly selective and specific anticancer activity on the uterine leiomyoma.

Female sexual hormones, estrogen and progesterone, are closely related to the growth of uterine leiomyoma [43,53]. Both estrogen and progesterone exert their physiological activities by binding with their specific nuclear receptors: The estrogen receptor (ER) and the progesterone receptor (PR). Previous studies showed that both ER and PR expression levels increased in uterine leiomyoma compared with normal myometrium [54,55]. Several ER-mediated genes have also been identified as having higher expression levels in uterine leiomyoma than in autologous myometrium [56]. Specifically, the over-expression level of aromatase P450 was identified in situ in uterine leiomyoma, which catalyzes androgens to estrogens, having a critical role in the promotion of leiomyoma growth [44,45]. ISL exhibits an estrogenic activity that has been reported to prevent breast cancer cell proliferation [57]. To better understand the physiological environment of uterine leiomyoma, we verified whether ISL treatment could inhibit the E2-induced growth of uterine leiomyomas. Consistent with previous findings, the results from this study show that E2 induced uterine leiomyoma and UtSMC cell growth. However, this E2-stimulated effect was inhibited by co-treatment of cells with E2 and ISL. Exogenous estrogen exposure, administered with contraceptives or menopausal hormone therapy, has been reported to be a factor increasing uterine leiomyoma incidence [58,59]. Our animal data also shows that the weight of the uterus increased with E2 treatment. This E2-stimulated effect was inhibited by co-treatment with E2 and ISL, which was observed to align with our in vitro outcomes. The serum E2 and P4 levels were decreased by co-treatment with E2 and ISL. E2-induced uterine myometrium proliferation was suppressed by co-treatment with E2 and ISL. Therefore, these results suggest that estrogen-regulated uterine leiomyoma cell proliferation could be suppressed by ISL treatment.

To explain the mechanism of ISL-induced inhibition of cell proliferation, the effect of ISL on cell cycle distribution was examined. We found that co-treatment of ELT3 cells with E2 and ISL induced cell cycle arrest at the G2/M phase, whereas the distribution of the sub-G1 phase increased. In agreement with our finding, Park and colleagues reported that ISL inhibits HeLa cervical cancer cell proliferation by inducing G2/M phase arrest and DNA damage [60]. We previously reported that ISL induced G2/M arrest by up-regulation of CDK2 protein expression and down-regulation of cyclin B1 protein expression in ovarian cancer [36]. MAPK signaling cascades are widely involved in the cell cycle, proliferation, survival, as well as in tumor development [46,61]. Activated ERK proteins have been reported to be involved in the promotion of cell survival through enhancing anti-apoptotic protein activation and down-regulating pro-apoptotic protein activation [62]. Additionally, it has been reported that p38 and JNK activation play important roles in crosstalk between autophagy and apoptosis [63]. In this present study, we found that co-treatment of ELT3 cells with E2 and ISL reduced ERK1/2 protein phosphorylation, whereas the phosphorylated levels of p38 and JNK were increased. Therefore, Hoechst 33342 nucleus staining was performed to confirm whether ISL induces DNA damage. The results show that the fluorescence intensity of Hoechst 33342 was increased by co-treatment of ELT3 cells with E2 and ISL. However, ISL treatment reduced the distribution of the S phase but did not increased the sub-G1 phase in UtSMC cells and did not alter the nucleus, as confirmed by Hoechst 33342 staining. These results imply that the cell death program could be induced by ISL treatment in ELT3 cells.

ISL has been reported to induce apoptosis via both intrinsic and extrinsic pathways in various cancer cells [22]. Hence, the effect of ISL on the apoptotic cell death program was investigated. In this study, we found that the population of apoptotic cells increased after co-treatment with E2 and ISL. The expression level of the apoptotic marker protein, cleaved PARP, increased, whereas the expression level of Bcl-2, an anti-apoptotic protein, decreased. In agreement with our results, Kim and colleagues reported that ISL induces uterine leiomyoma cell cycle arrest, the activation of caspase-3, and the down-regulation of Bcl-2, CDK2/4, and E2F protein expression levels [64].

The anti-apoptotic Bcl-2 family proteins Bcl-2 and Bcl-Xl are involved in anti-autophagy regulation via interaction with the Beclin 1 protein. Bcl-2 interacts with Beclin 1 to suppress autophagosome assembling, thereby inhibiting autophagy [47,48]. Therefore, we further examined whether autophagy signaling would be enhanced by ISL presence. In agreement with our results, Chen and colleagues found that downregulation of mTOR phosphorylation increased ISL-induced autophagy [65]. Inhibition of mTOR activity was shown to decrease ULK1 and Atg13 phosphorylation, which, in turn, reduced autophagosome formation [66]. However, a cytosolic form of LC3 I is conjugated to phosphatidylethanolamine to form LC3 II, which is recruited to autophagosomal membranes [67]. Our results also showed that total levels of LC3, an autophagosome marker, and the LC3 II/LC3 I ratio increased by co-treatment of ELT3 cells with E2 and ISL. These results suggest that ISL could induce autophagy pathway activation.

Extracellular matrix (ECM) accumulation is affected by growth factors, cytokines, and steroid hormones. An excessive ECM and accumulation around uterine leiomyoma cause the pathological development of leiomyoma [68]. Previously, we showed that ELT3 cells treated with fucoidan can reduce ECM-associated protein expression [69]. Matrix metalloproteinases (MMPs) and their regulation protein, tissue inhibitor of MMPs (TIMPs), are expressed in uterine leiomyoma for regulation of EMC remodeling [70]. In this present study, we showed that E2-induced levels of EMC-associated proteins and MMP-9 were reduced by the presence ISL, whereas the expression level of TIMP2 increased. Therefore, our results suggest that ISL could play a role in the regulation of ECM remodeling in uterine leiomyoma.

## 4. Materials and Methods

### 4.1. Cell Lines and Culture Conditions

The Eker rat-derived uterine leiomyoma ELT3 cell line was provided by Dr. Lin-Hung Wei (Department of Oncology, National Taiwan University Hospital, Taipei, Taiwan). The primary human uterine smooth muscle (UtSMC) cells were purchased from PromoCell (Heidelberg, Germany). UtSMC cells were isolated from the middle layer of the uterine myometrium and the short-tandem repeat DNA profiling was analyzed. Both cells were grown in Dulbecco’s Modified Eagle Medium/Ham’s F-12 Medium in a 1:1 ratio (DMEM/F-12; CAISSON Labs, Smithfield, UT, USA), supplemented with 10% fetal bovine serum (FBS; GIBCO, Grand Island, NY, USA), 100 units/mL penicillin (CORNING; Manassas, VA, USA), 100 μg/mL streptomycin, sodium bicarbonate (2.438 g/L, BioShop, Burlington, ON, Canada), and 4-(2-Hydroxyethyl)piperazine-1-ethanesulfonic acid (HEPES; 5.986 g/L; BioShop) in a humidified incubator (37 °C, 5% CO_2_). After resuscitation, ELT3 cells were sub-cultured with less than 20 passages, and UtSMC from 5–12 were used.

### 4.2. Cell Viability Assay

The effect of ISL on cell survival rate was analyzed using the MTT (3-[4,5-dimethyl-2-thiazolyl]-2,5-diphenyl-2H-tetrazolium bromide; Abcam, Cambridge, MA, USA) assay. At the end of incubation, the media were removed and replaced by serum-free culture medium with 0.5 mg/mL MTT and incubated for an additional 3 h. Subsequently, the media were removed. The crystal formazan in each well was dissolved in 100 μL dimethyl sulfoxide (DMSO; ECHO Chemical Co. Ltd., Taipei, Taiwan). The optical density was measured by using a VERSA Max microplate reader (Molecular Devices, San Jose, CA, USA) at 570 nm and 630 nm as a reference wavelength.

### 4.3. Crystal Violet Staining

Crystal violet staining was also performed to observe the cell survival rate. At the end of incubation, the media were removed and stained with 200 μL per well crystal violet solution (0.5%; Sigma-Aldrich, St Louis, MO, USA) for 20 min at room temperature. Then, the crystal violet solution was removed and washed twice with 1× PBS. The plate was air dried and photographed. Crystals were dissolved in 500 μL DMSO (ECHO Chemical Co. Ltd.). Then, 100 μL of the 500 μL well-dissolved solution was transferred into a 96-well plate. The optical density was measured using VERSA Max microplate reader (Molecular Devices) at a wavelength of 630 nm.

### 4.4. Cell Counting

At the end of incubation, cell morphologies were photographed using a light microscope (Olympus, Tokyo, Japan). Then cells were detached using 0.05% trypsin-EDTA reagent and culture media were collected in 15 mL tubes and then centrifuged to harvest a cell pellet. The supernatant was removed and the cell pellet resuspended in 1 mL 1× PBS, and then the suspended cells were mixed with 0.4% trypan blue solution. The numbers of cells were counted using a hemocytometer under a microscope. In addition, ADAM-MC automatic cell counter (NanoEntTek Inc., Waltham, MA, USA) was used to confirm the cell number. The procedure was performed according to the manufacturer’s protocols.

### 4.5. Flow Cytometry Analysis of Cell Cycle Distribution and Apoptosis

The ELT3 and UtSMC cells were seeded in 2.5 × 10^5^ and 3 × 10^5^ cells in 6-cm culture dishes, respectively. After attaching overnight, cells were rendered quiescence in DMEM/F-12 medium supplemented with 0.4% charcoal-stripped FBS for 24 h, and then treated with estradiol (E2; 100 nM) alone or co-treated with E2 (100 nM) and ISL (10 or 40 μM) in DMEM/F-12 medium containing 10% charcoal-stripped FBS for an additional 48 h. At the end of incubation, the cells were detached by trypsinization and collected together with culture media into 15 mL tubes. After centrifugation, the supernatant was removed, and the cell pellets were resuspended in 1 mL 1× PBS and then mixed with 70% ethanol at −20 °C for at least 2 h. Next, the cell pellets were centrifuged and resuspended in propidium iodide (PI) mixture solution (2 mg DNAse-free RNAse A and 0.4 mL of 500 μg/mL PI was added to 10 mL of 0.1% Triton X-100 in PBS) at room temperature for 30 min. The density of fluorescence in cell samples was analyzed using BD FACSCalibur flow cytometer (BD Biosciences, San Jose, CA, USA).

Both ELT3 and UtSMC cells (3 × 10^5^ cells) were treated with E2 (100 nM) alone or co-treated with E2 (100 nM) and ISL (10 or 40 μM) in DMEM/F-12 medium containing 10% charcoal-stripped FBS for 48 h. The apoptosis analysis was performed using fluorescein isothiocyanate (FITC) Annexin V Apoptosis Detection Kit I (BD Biosciences). The cell pellets were harvested as indicated above. Finally, cell pellets were resuspended in Annexin V-FITC and PI solution and then incubated at room temperature for 15 min, followed by flow cytometric analysis (BD Biosciences).

In the cell cycle and apoptosis assay, a minimum of 10,000 cells per sample were collected and further analyzed using CellQuest software (version 5.1, BD Biosciences).

### 4.6. Hoechst 33342 Staining

All procedures were performed according to the manufacturer’s protocol of NucBlue Live ReadyProbes Reagent Kit (Thermo Fisher Scientific, Waltham, MA, USA) and incubated for 20 min, protected from light. Cell images were photographed at 200× magnifications using an EVOS^®^ microscope (Thermo Fisher Scientific).

### 4.7. Protein Preparation and Western Blot Analysis

The total proteins were extracted, and Western blot analyses were performed as previously described [69]. Briefly, the cell and tissues were lysed with RIPA buffer fortified with a protease inhibitor (Roche, Basel, Switzerland) and a phosphatase inhibitor (Roche). Protein concentrations were measured using the BCA Protein Assay Kit (T-Pro Biotechnology, New Taipei City, Taiwan). Next, 40 μg of the samples were loaded into 7.5 or 12% sodium-dodecyl sulfate (SDS)-polyacrylamide gels, transferred with (BIO RAD) system, and assayed with anti-PARP (Cell Signaling, Boston, MA, USA), anti-CDK4 (Cell Signaling), anti-Bcl-2 (Cell Signaling), anti-Bax (Cell Signaling), anti-phosph-mTOR (Cell Signaling), anti-mTOR (Cell Signaling), anti-phosph-ULK1 (Cell Signaling), anti-ULK1 (Cell Signaling), anti-phosph-ERK1/2 (Cell Signaling), anti-ERK1/2 (Cell Signaling), anti-phosph-p38 (Cell Signaling), anti-p38 (Cell Signaling), anti-phosph-JNK (Cell Signaling), anti-JNK (Cell Signaling), anti-p62 (Abcam, Cambridge, MA, USA), anti-LC3 (Abcam), anti-cyclin D1 (Abcam), anti-GAPDH (Proteintech, Rosemont, IL, USA), anti-α-SMA (GeneTex, Irvine, CA, USA), anti-fibronectin (Santa Cruz, Biotechnology, Santa Cruz, CA, USA), anti-vimentin (Santa Cruz), anti-β-actin (Santa Cruz), anti-collagen type 1 (GeneTex), and anti-MMP 9 (Santa Cruz) antibodies. The membranes were then incubated with horseradish peroxidase-conjugated goat anti-mouse and/or rabbit antibodies (Jackson ImmunoResearch Laboratories, West Grove, PA, USA) for 1 h. A Biotinylated-Avidin solution kit (Vector Laboratories, Burlingame, CA, USA) was used to enhance phopho-p38 and phosph-JNK signaling. The visual signal was captured by an Amersham Imager 600 system (GE Healthcare Life Sciences, Pittsburgh, PA, USA). The band densities were determined as arbitrary absorption units using the Image-J software program (version 1.45s; National Institutes of Health, Bethesda, MD, USA).

### 4.8. Immunofluorescent Staining

The immunofluorescent staining was performed as previously described [38]. Briefly, the cells were fixed with 4% paraformaldehyde, blocked by 5% BSA in 1× TBST, and assayed with anti-LC3 II antibody (Abcam). Finally, the sections were coverslipped with ProLong Gold antifade mounting solution containing DAPI nuclear stain (Thermo Fisher Scientific), and then an EVOS^®^ microscope (Thermo Fisher Scientific) was used to image the immunofluorescent signals.

### 4.9. Animal Experiments

All animal studies were conducted according to the protocols approved by the Institutional Animal Care and Use Committee (IACUC) of Taipei Medical University (Permit number: LAC-2017-0266). In this study, four- to five-week-old ICR(CD-1) female mice were purchased from BioLASCO (Taipei, Taiwan). The mice were housed in 12 h artificial illumination and temperature (22 ± 2 °C) controlled room. Food and water were provided ad libitum.

The process of mouse uterine hyperplasia animal model generation is illustrated as Figure 7A. After adaption for 1 week, mice were randomly divided into five groups: (i) mice were administered with DMSO via subcutaneous injection as vehicle control group once daily for 8 weeks; (ii) mice were treated with E2 (0.3 mg/kg/day) via subcutaneous injection once daily for 8 weeks as the model control group; mice were subcutaneous injected with E2 (0.3 mg/kg/day) combined with ISL at low concentration (1 mg/kg/day, ip injection) (iii) or high concentration (5 mg/kg/Day, ip injection) (iv) three times a week for 8 weeks; and (v) the positive group was generated by co-treatment of mice with E2 (0.3 mg/kg/day, subcutaneous injection) and tamoxifen (TAM; 0.25 mg/kg/day, ip injection) for three times a week for 8 weeks.

At the end of experiment, the mice were euthanized using an anesthetic mixture solution (1 mL Zoletil and 0.1 mL rompun in 3.9 mL normal saline). Under aseptic conditions, blood samples were collected by heart puncture. Subsequently, uteri were quickly isolated and immediately cut into two pieces, with one stored at −80 °C and the other one fixed in 10% formalin for further analysis.

### 4.10. ELISA of Serum Estradiol and Progesterone

The concentrations of serum E2 and P4 were measured using an E2 ELISA kit (Cayman Chemical, Ann Arbor, MI, USA) and a P4 ELISA kit (Cayman Chemical), respectively. All procedures were performed according to the manufacturer’s protocols. The absorbance values of these two hormone kits were measured using VERSA Max microplate reader (Molecular Devices) at 420 nm.

### 4.11. H&E Staining

Uterine tissues were fixed in 10% formalin for 24 h. Tissues were dehydrated and embedded in paraffin, then sliced into 3–5 μm cross-sections. Then, tissue sections were stained with hematoxylin and eosin (H&E) solutions. All the procedures were completed at the animal experimental center of Taipei Medical University (Taipei, Taiwan). The EVOS^®^ microscope (Thermo Fisher Scientific) was employed for imaging.

### 4.12. Immunohistochemistry (IHC) Staining

Paraffin-embedded uterine tissues were sliced into 3–5 micrometer cross sections, then deparaffinized, rehydrated, and assayed with anti-α-SMA (1:200; GeneTex). The IHC staining procedures were completed by the Bio-Check Laboratories LTD (Taipei, Taiwan). The EVOS^®^ microscope (Thermo Fisher Scientific) was employed for imaging.

### 4.13. Statistical Analysis

All quantitative results are expressed as mean ± standard error of the mean (SEM) analyzed using Prism version 6.0 software (GraphPad, San Diego, CA, USA). The statistically significant difference was determined by one-way analysis of variance (ANOVA) for all groups. The Student’s unpaired *t*-test was used for comparison between two groups. Significance was accepted at * *p* < 0.05 and ** *p* < 0.01.

## 5. Conclusions

**Figure 9:**
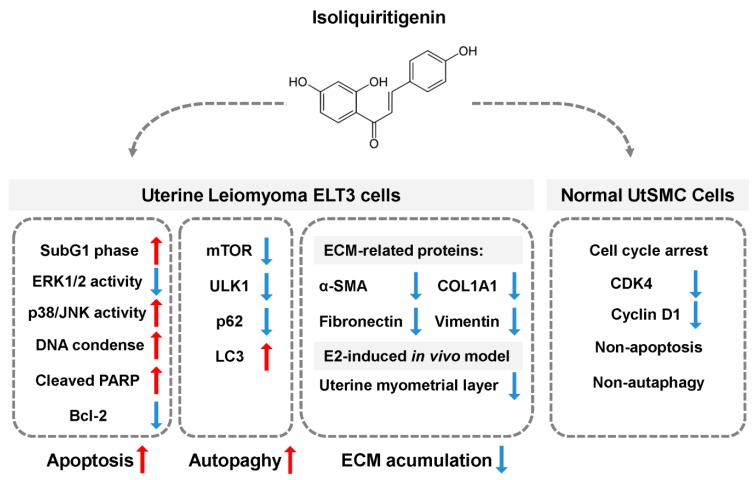
Schematic representation of the inhibitory effect of ISL on the proliferation of uterine leiomyoma through promoting the cell death program. Treatment of uterine leiomyoma ELT3 cells with ISL induced apoptosis and activated autophagy cell death through regulation of MAPK pathway. The expression of extracellular matrix (ECM)-associated proteins was decreased after ISL treatment in both in vitro and in vivo models. In addition, ISL treatment suppressed primary human uterine smooth muscle UtSMC cells growth via arresting the cell cycle.

In conclusion, the results of this study show that isoliquiritigenin (ISL) exerts an effective and higher selection on the inhibition of estrogen-induced uterine leiomyoma growth both in vitro and in vivo. ISL inhibits cell cycle arrest and triggers apoptosis and autophagy cell death programs in uterine leiomyoma cells. In contrast, ISL can only suppress cell cycle progression in the normal uterine smooth muscle cells. ISL reduced estrogen-induced uterine myometrium hyperplasia through down-regulation of extracellular matrix-associated proteins expression. Overall, we propose a schematic model of the mechanism underlying ISL-induced growth of uterine leiomyoma cells (Figure 9). Based on the results of this study, we propose that ISL might be a new option for the treatment of human uterine leiomyoma.

## Supplementary Materials

The following are available online at https://www.mdpi.com/2072-6694/11/8/1131/s1, Figure S1: ISL treatment induced DNA damage in ELT3 cells, Figure S2: ISL treatment decreased the expression of ECM-associated proteins.
