# Supplementary material for: Isoliquiritigenin Suppresses E2-Induced Uterine Leiomyoma Growth through the Modulation of Cell Death Program and the Repression of ECM Accumulation
Source: Cancers (Basel). 2019 Aug 7;11(8):1131. doi: 10.3390/cancers11081131 (PMC6721550; doi:10.3390/cancers11081131)
Supplement: Supplementary file 1 [file cancers-11-01131-s001.pdf]

Supplementary Materials: Isoliquiritigenin Suppresses E2-Induced Uterine Leiomyoma Growth through the Modulation of Cell Death Program and the Repression of ECM Accumulation

Po-Han Lin, Hsiang-Lin Kung, Hsin-Yuan Chen, Ko-Chieh Huang and Shih-Min Hsia

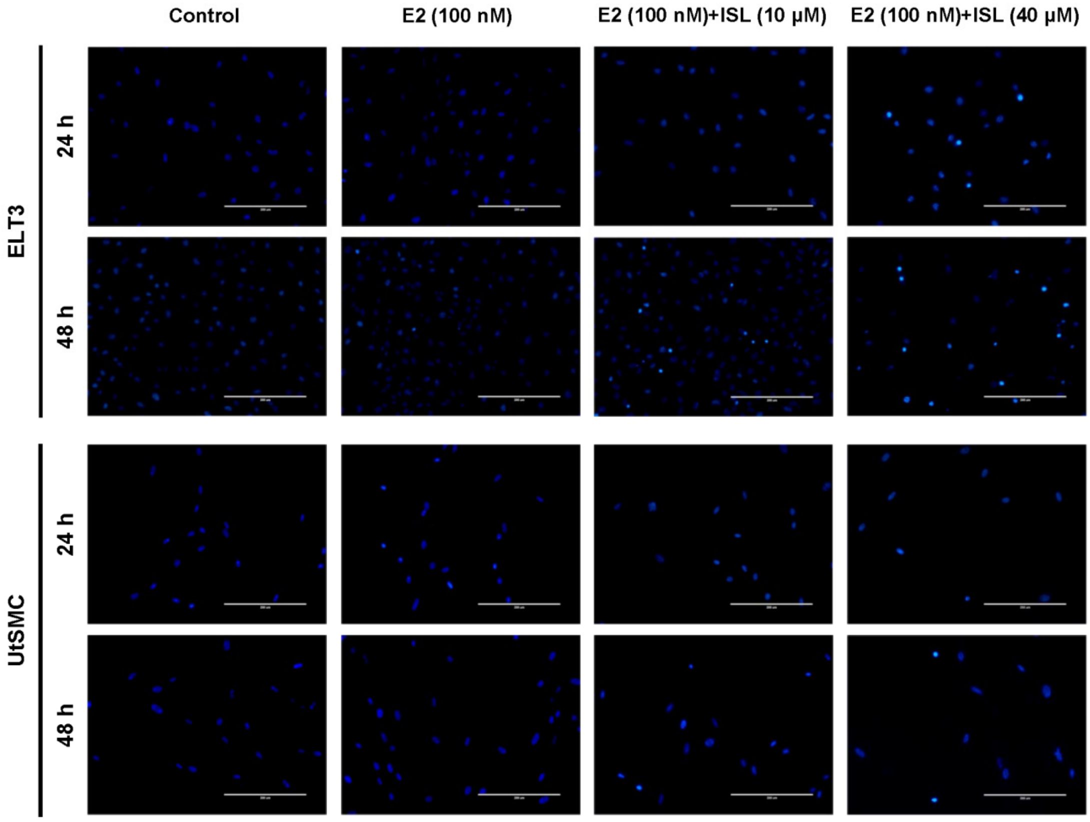

Figure S1. ISL treatment induced DNA damage in ELT3 cells. ELT3 and UtSMC cells (5 × 10^5 cells) were treated with E2 (100 nM) alone or a combination of E2 and ISL (10 or 40 μM) for 24 and 48 h. Cells were stained with Hoechst 33342 solution. (magnification 200×; Scale bar = 200 μm).

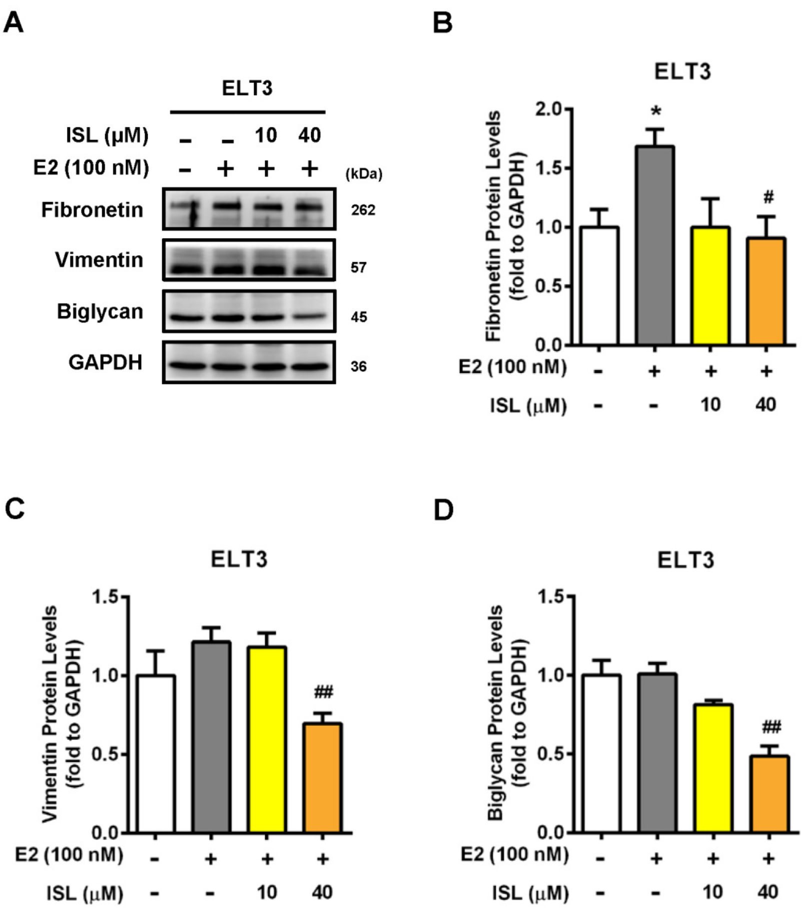

Figure S2. ISL treatment decreased the expression of ECM-associated proteins. (**A**) ELT3 cells were treated with E2 alone or combination with E2 and ISL (10 or 40 μM) for 48 h. At the end of incubation, cells were lysed and then ECM-associated proteins were analyzed by Western blot. The expression of (**B**) fibronection, (**C**) vimentin, and (**D**) biglycan were normalized to GAPDH expression in ELT3 cells. Data are represented as means ± SEM (n = 3). * $p < 0.05$ as compared with the control group. # $p < 0.05$, ## $p < 0.01$ as compared with the E2-treated group.

© 2019 by the authors. Licensee MDPI, Basel, Switzerland. This article is an open access article distributed under the terms and conditions of the Creative Commons Attribution (CC BY) license (http://creativecommons.org/licenses/by/4.0/).
